# Viewing Majorana Bound States by Rabi Oscillations

**DOI:** 10.1038/srep11686

**Published:** 2015-07-08

**Authors:** Zhi Wang, Qi-Feng Liang, Dao-Xin Yao, Xiao Hu

**Affiliations:** 1 School of Physics and Engineering, Sun Yat-sen University, Guangzhou 510275, China; 2 International Center for Materials Nanoarchitectonics (WPI-MANA), National Institute for Materials Science, Tsukuba 305-0044, Japan; 3 Department of Physics, Shaoxing University, Shaoxing 312000, China

## Abstract

We propose to use Rabi oscillation as a probe to view the fractional Josepshon relation (FJR) associated with Majorana bound states (MBSs) expected in one-dimensional topological superconductors. The system consists of a quantum dot (QD) and an rf-SQUID with MBSs at the Josephson junction. Rabi oscillations between energy levels formed by MBSs are induced by ac gate voltage controlling the coupling between QD and MBS when the photon energy proportional to the ac frequency matches gap between quantum levels formed by MBSs and QD. As a manifestation of the Rabi oscillation in the whole system involving MBSs, the electron occupation on QD oscillates with time, which can be measured by charge sensing techniques. With Floquet theorem and numerical analysis we reveal that from the resonant driving frequency for coherent Rabi oscillation one can directly map out the FJR cos(*π*Φ/Φ_0_) as a signature of MBSs, with Φ the magnetic flux through SQUID and Φ_0_ = *hc*/2*e* the flux quantum. The present scheme is expected to provide a clear evidence for MBSs under intensive searching.

Majorana bound states (MBSs) in topological superconductors become the focus of many recent researches^1^,^2^,^3^,^4^,^5^,^6^. These bizarre quasi-particles are equal quantum superposition of electrons and holes, and obey the non-Abelian statistics^7^,^8^. Pairs of MBSs can be used to constitute topological quantum bits (qubits), and several ingenious ideas have been proposed to carry out information processing by braiding MBSs^7^,^9^,^10^,^11^. Decoherence suffered by other approaches to realize qubits is expected to be suppressed since MBSs themselves are charge neutral and the qubits formed by MBSs are nonlocal in space, which make them robust to local, accidental perturbations such as electromagnetic noises^3^. MBSs have been predicted in various topological superconducting systems, including superconductor-topological insulator interfaces^12^, spin-orbit coupled semiconductor and superconductor composites^13^,^14^,^15^, nanotube-superconductor devices^16^. In particular, spin-orbit-coupled semiconductor nanowires in proximity to *s*-wave superconductors have been investigated experimentally^17^,^18^,^19^,^20^,^21^,^22^,^23^, because they are relatively easy to manufacture and are described by a simple model Hamiltonian^2^. In this regard, it is also noticed that several works appear very recently on ferromagnetic nanowire put proximately to substrate *s*-wave superconductor^24^.

The unconventional properties of MBSs as opposed to the well-known boson and fermion make them difficult to be captured in experiments by conventional techniques. Therefore, how to confirm their existence has been one of the topmost issues ever since the theoretical proposal of MBS in condensed matter systems^25^,^26^,^27^. As a major distinct property, MBSs transport supercurrent through a junction between two topological superconductors in terms of the *fractional* Josephson relation (FJR) *I* ~ ±*E* sin(*ϕ*/2), where ±*E* are energies of the two Andreev bound states formed by MBSs and *ϕ* is the gauge invariant phase difference between the two superconductors^2^,^28^. Naively, this looks to indicate a critical Josephson current with 4*π* period. However, in realistic systems the wave-functions of MBSs at junction overlap with the MBSs at two far ends of nanowires, which opens a small gap between the two branches when they cross each other upon phase variation, known as MBS poisoning^29^. Therefore, it is impossible in principle to detect the 4*π* period in the dc critical Josephson current, since in adiabatic processes of phase tuning the system should relax into the state of lower energy, which mixes the two branches ±*E* and reduces the period of current phase relation to the conventional value of 2*π*. Large voltages may rotate the phase quickly to avoid the relaxation process at the band crossing point. Suppression of odd-number Shapiro steps was reported which apparently looks consistent with the FJR^18^. However, suppression of odd-number Shapiro steps can happen even in conventional SNS junctions constructed by *s*-wave superconductors, and thus cannot be taken as a unique signal of MBSs^30^. Some other ideas were proposed to get possible signals from MBSs, such as to measure current noises or electromagnetic radiations with frequency half to the conventional Josephson relation^31^,^32^,^33^, and to use current bias for detection of Shapiro steps^34^. Despite intensive efforts^27^, a compelling evidence for MBSs is still missing so far.

In this work, we propose to directly view the FJR using Rabi oscillations between energy levels formed by MBSs. As schematically shown in Fig. 1, our setup is constructed by a Majorana rf-SQUID and a nearby quantum dot (QD), where an ac gate voltage is applied to tune the coupling between QD and MBS periodically in time; the magnetic flux through SQUID modulates the phase difference between the two MBSs at junction. The time evolution of quantum states including MBSs and QD is investigated by the Floquet theorem and numerical techniques. Intriguingly we find that a pair of resonant driving frequencies for coherent Rabi oscillations depend individually on the magnetic flux in the form of FJR cos(*π*Φ/Φ_0_) associated with the MBSs. Experimentally the Rabi oscillations can be detected by sensing the electron occupation on QD with well established techniques^35^.

Since the Josephson junction in the present rf-SQUID is induced by a tunneling barrier from local gate voltage, other possibilities to generate FJR, such as those in ballistic SNS junctions and long junctions^29^,^36^ can be excluded. Interactions between MBSs in finite nanowires, the source of MBS poisoning, have been taken into consideration from the beginning, and dynamic transformations among MBS levels are treated adequately by Floquet theorem. Therefore, the present scheme is expected to be able to provide an unambiguous evidence for MBSs in one-dimensional topological superconductors.

## Results

### MBS-SQUID and Rabi oscillation

**Figure 1:**
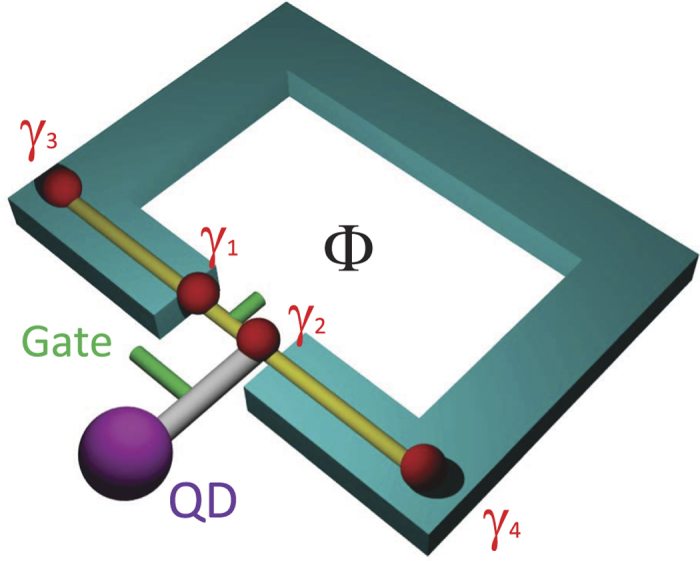
Schematic setup of a hybrid system constructed by a topological rf-SQUID and a quantum dot. The quantum dot is connected to the Majorana bound states in the SQUID with the coupling strength tunable by an ac gate voltage.

The Majorana SQUID is formed by a Josephson junction with MBSs at both sides of the junction as shown in Fig. 1. For example, one can put a semiconductor nanowire with spin-orbit coupling on a conventional *s*-wave superconductor with ring shape. When the chemical potential and Zeeman field are tuned appropriately, the semiconductor nanowire enters the topological superconducting phase due to proximity effect with MBSs accommodating at the ends^13^,^15^,^17^. A tunneling barrier is introduced at the center of the nanowire, for example, by a dc gate voltage, resulting in a Josephson junction with two MBSs additionally. The Hamiltonian for low-energy physics of the system is given in terms of MBSs^2^,^9^
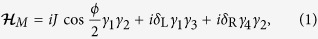
where the first term is the fractional Josephson energy associated with the two MBSs *γ*_1_ and *γ*_2_ at the junction with *J* the energy integral of the junction, *ϕ* = 2*π*Φ/Φ_0_ the phase difference across the junction induced by the magnetic flux Φ through SQUID with Φ_0_ the flux quantum; *γ*_3_ and *γ*_4_ are the two MBSs at the two ends of the wire, as illustrated in Fig. 1; *δ*_L_ and *δ*_R_ are couplings among MBSs due to small wave-function overlaps in the two segments.

The QD is prepared in such a way that only one level is energetically relevant, and thus its state is described by 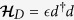 with *d*^†^ the electron creation operator and 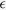 the electron occupation energy. The QD is tunneling connected to the MBSs at the Josephson junction with 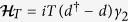, where *T* is the coupling strength controlled by an ac gate voltage (see Fig. 1) and changes with time periodically *T* = *T*_0_ + 2*T*_1_cos Ω*t*. For clarity, we treat explicitly the case that QD is coupled to one of the two junction MBSs, the physics discussed below is valid for a general case where QD is coupled simultaneously to the two junction MBSs (see Supplement for details).

The dynamics of the present system is described by the time-dependent Schrödinger equation,
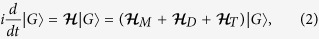
where *ħ* = 1 is taken. Defining two fermionic operators with the four MBSs 
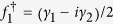
 and 
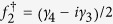
, the basis functions for Hamiltonian 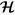 can be set as 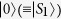, 
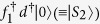
, 
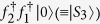
, 
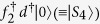
, 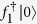, 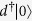, 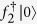, and 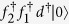, where 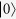 is the vacuum for operators *f*_1_, *f*_2_ and *d*. In this basis, the Hamiltonian 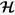 is an 8 × 8 matrix. With the conservation of parity concerning the number of fermionic particles upon on application of gate voltage and Cooper pair tunneling, Hamiltonian 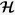 is block-diagonal. Without losing generality, we hereafter focus on the even-parity subspace of the system. The Schrödinger equation (2) then reads
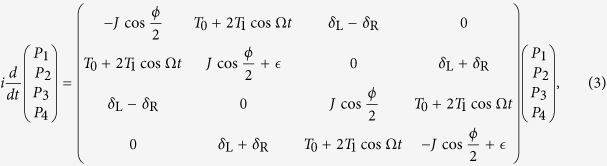
with 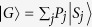.

We initialize the system by measuring the electron occupation on the QD. Let us start from the vacant QD, which corresponds to a superposition of 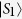 and 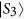 with 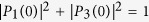.

**Figure 2:**
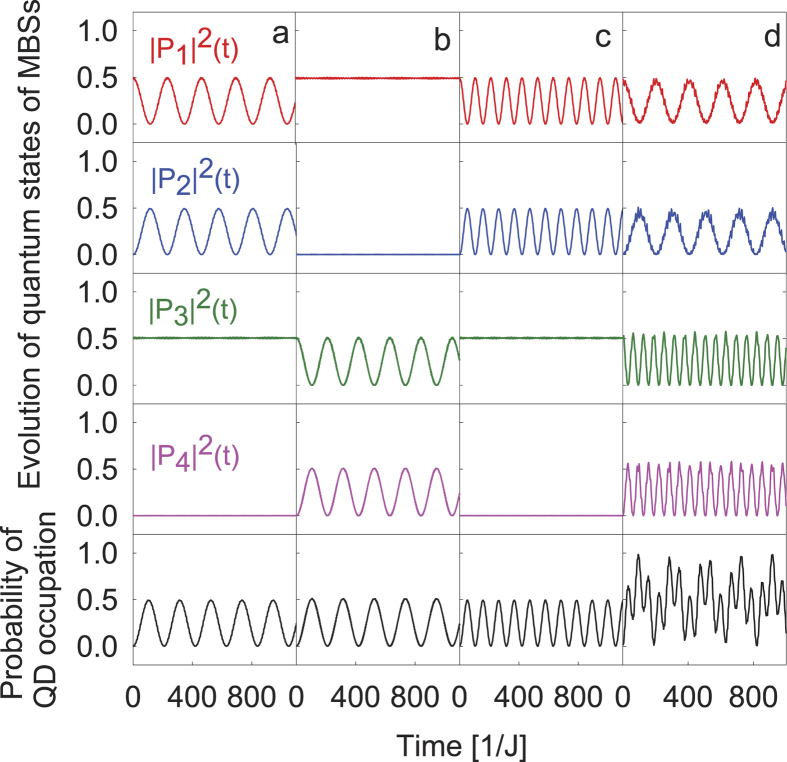
Time evolution of the weights for the quantum states and the QD occupation probability 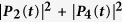
**, with the initial condition** 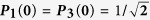, (**a**) for Φ = 0 and driving frequency Ω = 2.402 *J* and *T*_1_ = 0.015 *J*, (**b**) for Φ = 0 and Ω = 1.602 *J* and *T*_1_ = 0.015 *J*, (**c**) for Φ = 0 and Ω = 2.402 *J* and *T*_1_ = 0.03 *J*, and (**d**) for Φ = 0.436 Φ_0_ and Ω = 0.8 *J* and *T*_1_ = 0.015 *J*, respectively. Other parameters are *T*_0_ = 0.05 *J*, *δ*_L_ = 0.02 *J*, *δ*_R_ = 0.005 *J*, and 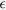 = 0.4 *J*. *ħ* = 1 is taken through the paper.

The time evolution of the system can be obtained in terms of the weights *P*_*i*_(*t*) with 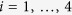 by integrating Eq. (3). Typical time evolutions of the quantum state are given in Fig. 2. It is clear that the tunneling interaction *T* stimulates resonant 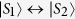 and 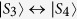 oscillations and associated oscillations in the occupation probability on QD at two different frequencies Ω for a given magnetic flux Φ, as shown in Fig. 2(a,b). When the amplitude of ac component *T*_1_ in *T* is doubled, the frequency of oscillation is doubled as shown explicitly in Fig. 2(c), which evidences Rabi oscillations in the system.

### Floquet theory

In order to understand the Rabi oscillations better, we analyze the system in terms of the Floquet theorem^37^. Since the system is driven by the ac voltage, the solution of Eq. (3) should be given in the form 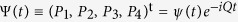, with *ψ*(*t*) a periodic function 
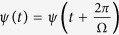
, and *Q* the eigenvalue to be determined. The Fourier components of the wave-function *ψ*(*t*) and the Hamiltonian are given as
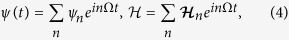
with
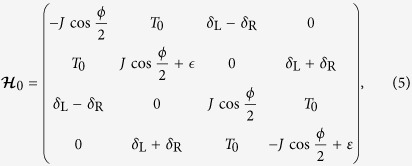

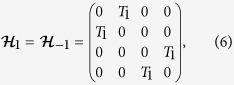
and all other matrices zero in the present case. Plugging them into Eq. (3), we arrive at the following static secular equation^37^,
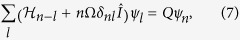
with 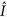 the 4 × 4 identity matrix. Defining a vector 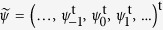, we obtain the time-independent Floquet Hamiltonian,
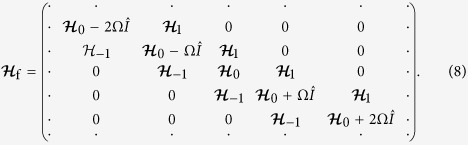


Now the original system of four states with time-periodic Hamiltonian 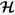 in Eq. (3) is transformed into a system with static Floquet Hamiltonian 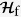. The diagonal part of the Floquet Hamiltonian is built by infinite 4 × 4 blocks, with each block formed by the time-invariant part of the original Hamiltonian 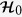 adding energy quanta *n*Ω associated with integer number of “photons”; any two diagonal blocks with one-photon difference are connected by an off-diagonal 4 × 4 block given by the amplitude of time-periodic part in the original Hamiltonian. The basis states for the Floquet Hamiltonian 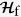 are the Floquet states 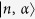, with *α* referring to the four states 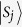 (*j* = 1, 2, 3 and 4) and *n* to the Fourier component.

Within the Floquet theory^37^, it has been shown that the transition probability between two quantum states 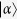 and 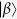 can be expressed as a summation of those between corresponding Floquet states 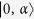 and 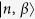

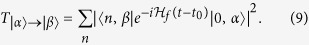


Therefore, the problem of evaluating the transition probability between two quantum states governed by time-dependent Hamiltonian is reduced to a corresponding one with time-independent Floquet Hamiltonian, with the latter being a conventional problem in quantum mechanics.

For example, let us consider the probability of the transition between quantum states 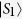 and 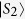. With a second-order perturbation treatment on the Floquet Hamiltonian Eq. (8), we obtain the effective 2 × 2 Hamiltonian (see Supplement for details) for transition between Floquet states 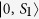 and 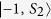

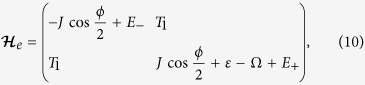
with energy shift 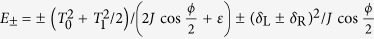. Starting from 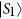, the system evolves with time according to
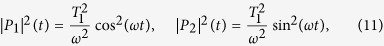
with 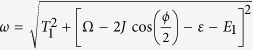 and 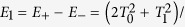 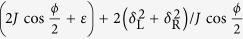. When the photon energy of the driving ac voltage fills the energy gap between 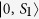 and 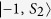, a coherent Rabi oscillation between the two levels appears characterized by the maximal oscillation amplitude in the occupation probability, consistent with the numerical result in Fig. 2(a,c). In the same way, one has the contribution from the Floquet state 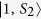. The spectrum for coherent Rabi oscillation among 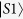 and 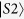 is then given by
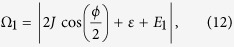


With the same procedure, we can obtain the Rabi oscillation between 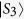 and 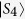 under the driving frequency
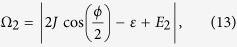
with 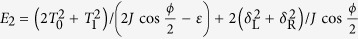, consistent with Fig. 2(b).

Equations (12) and (13) are the main results of this work. We emphasize that the resonant driving frequencies Ω_1_ and Ω_2_ for coherent Rabi oscillations vary with the applied magnetic flux in the way of cos(*π*Φ/Φ_0_), characterizing the FJR, which is an intrinsic feature of MBSs. Since the electron occupation on QD in state 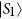 is different from that in state 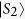 (same for 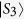 vs 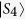), the Rabi oscillation of the whole system manifests itself as oscillation in electron occupation on QD, which is an important merit of our setup. Electron occupation on QD can then be detected experimentally by advanced techniques such as quantum point contact charge detector^35^.

**Figure 3:**
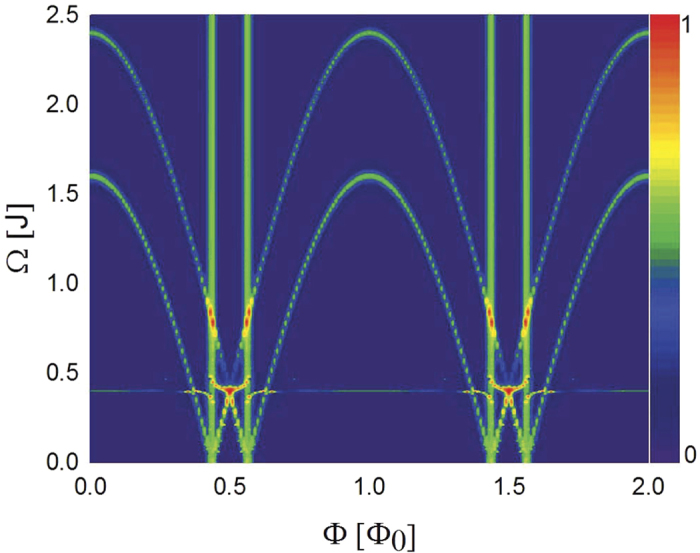
Driving frequency Ω for coherent Rabi oscillation as a function of applied flux Φ. The color is for the strength of Rabi oscillation measured by the peak-dip difference in the oscillation of the occupation probability on QD. The initial state is presumed as an equal weight of 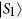 and 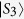 associated with empty QD. Parameters are the same as Fig. 2 except for *T*_1_ = 0.015 *J*.

In Fig. 3 we map out the full spectrum of resonant driving frequency for Rabi oscillations by sweeping the magnetic flux in the SQUID, derived from the numerical integration of Eq. (3). The two curves agree exactly with the analytic results Eqs. (12) and (13) for Rabi oscillations. There are four vertical lines in Fig. 3 at  = ±2*J* cos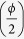. It is easy to see from Eq. (5) that 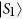 and 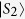 or 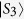 and 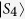 are degenerate in energy irrespective of driving frequency. In these cases, the simple treatment of Floquet theorem based on 2 × 2 matrix breaks down, where the denominators in energy shifts *E*_±_ (and thus *E*_1,2_) in Eqs. (12) and (13) become zero. The behavior of the system is however described accurately by numerical integration as shown in Fig. 2(d). There is a horizontal line at 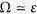 in Fig. 3, at which two Rabi oscillations 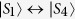 and 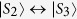 take place simultaneously (see Supplement for details).

## Discussions

As seen in Fig. 3 the period of resonant frequencies for coherent Rabi oscillations is Φ_0_, or equivalently 2*π* in phase, since the two cosine branches cross each other at Φ_0_/2 in a symmetric way. We emphasize that, despite of the overall 2*π* periodicity, the FJRs associated with MBSs are detectable by Rabi oscillations from the detailed magnetic-flux dependence of the two cosine curves (see also Eqs. (12) and (13)). In addition to the fractional Josephson coupling induced by the MBSs, there may be a conventional one proportional to cos*ϕ*. However, since the conventional Josephson coupling is associated with virtual processes composed by two steps involving states above superconducting gap, the relevant quasiparticle excitations exhibit higher energies as compared with the states formed by MBSs with weak tunneling coupling. Possible Rabi oscillations associated with these high-energy states and characterized by flux dependence cos*ϕ* should appear at higher frequencies and can be separated from the ones by MBSs addressed in the present work.

Experiments designed to detect quantum properties based on Rabi oscillation suffer usually damping of quantum coherence, and the present device shares the same difficulty. Since damping processes depend in quantitative ways on details of implementation of the setup, a general discussion is difficult at this stage, which would become a future work. We notice, however, that possible transitions to continuum spectra over superconducting gap can be suppressed in the present setup by lowering operation temperature. This is an advantage of the present approach based on Rabi oscillation over other proposals using ac Josephson effect, where a bias voltage along the junction direction is necessary which induces excitations to higher energies and reduces quantum coherence.

The Rabi oscillations revealed in the present work can be measured by sensing the charge on QD by well established techniques, such as quantum point contact charge detector^35^ which has been shown to be able to measure a single electron on QD. Therefore, the present proposal is expected accessible experimentally. Since there is no way to control the relative weights *P*_1_(0) and *P*_3_(0) although 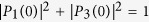 is initialized, the curves for oscillations of QD occupation probability shown in Fig. 2 should be replaced by envelope curve for experimental data with the same frequency (see Supplement for details).

The typical proximity induced superconducting gap in the semiconductor nanowire is around 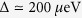
17, which sets the largest energy scale of the present system. The Josephson energy *J* should be one order smaller than the gap in magnitude and thus 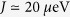; the tunneling energy *T* between QD and MBSs is considered to be in the same order or smaller than *J*; the MBS coupling energy *δ* is exponentially small for long enough wires. With these parameters, the driving frequencies for coherent Rabi oscillations are estimated in the range of GHz which can be achieved by electric circuits^38^, and the operating temperature is to be controlled to below 50 *mk*. Improvements in materials and interfacial treatments will relax the condition for temperature. The present scheme is also applicable to detect MBSs realized in systems composed by topological insulator and superconductor^39^.

## Methods

The model Hamiltonian consists of three parts, namely the coupling between two MBSs in the form of FJR at the Josephson junction of SQUID, a coupling between QD and junction, and small but finite interactions from two additional end MBSs which describe the MBS poisoning. We show that the low-energy physics of the system can be well described by a 4 × 4 Hamiltonian periodic in time when an ac voltage is applied at the gate between QD and MBSs at junction. The time-periodic Hamiltonian is then solved both by the Floquet theory analytically and in a numerical way. Full spectra of driving frequency for coherent Rabi oscillation are then mapped out upon tuning the magnetic flux applied through the SQUID, which can be detected experimentally by sensing the charge occupation on QD. The FJR associated with MBSs can be seen directly from the individual spectrum, even though the overall period is Φ_0_ in magnetic flux or 2*π* in phase.

## Additional Information

**How to cite this article**: Wang, Z. *et al.* Viewing Majorana Bound States by Rabi Oscillations. *Sci. Rep.* **5**, 11686; doi: 10.1038/srep11686 (2015).

## Supplementary Material

Supplementary Information
