# Supplementary material for: Viewing Majorana Bound States by Rabi Oscillations
Source: Sci Rep. 2015 Jul 8;5:11686. doi: 10.1038/srep11686 (PMC4495447; doi:10.1038/srep11686)
Supplement: Supplementary Information [file srep11686-s1.pdf]

Supplementary Material for "Viewing Majorana Bound States by Rabi Oscillations"

Zhi Wang, Qi-Feng Liang, Dao-Xing Yao and Xiao Hu

(April 1, 2015)

Model

Here we show the details for building the minimum model to describe our setup. The SQUID of the setup is build by a circled conventional superconductor with a straight spin-orbit coupling nanowire on top of it, as shown in Fig. 1 of the main text. The proximity effect induces a gap in the wire, making it superconducting. Under an appropriate magnetic field, the nanowire enters into a topological state and becomes a topological superconductor. There are two domain walls between the topological superconducting wire and the trivial superconducting base. A gate voltage is applied in the center of the wire, producing a well-defined tunneling junction. For convenience we call the two parts of nanowire as left and right segments. The critical current of this junction is small enough, so that the superconducting phase change across this junction is equal to the magnetic flux through the SQUID. Meanwhile, the nanowire is much smaller than the base, that the superconducting phase on the wire can be safely assumed to be the same within the segments. The low-energy physics of the system can be described by four Mjorana bound states (MBSs), two locating at the junction, and the other two at the domain walls. From the work of Kitaev[1], the MBSs at the junction are defined as

$$\gamma_1 = e^{i\frac{\theta_L}{2}}\hat{c}_1 + e^{-i\frac{\theta_L}{2}}\hat{c}_1^\dagger, \quad \gamma_2 = -ie^{i\frac{\theta_R}{2}}\hat{c}_2 + ie^{-i\frac{\theta_R}{2}}\hat{c}_2^\dagger \tag{S1}$$

where $\theta_{L,R}$ are the superconducting phase of the left/right segment, $\hat{c}_{1,2}$ are the local fermion operators at the two sides of the junction. Similarly, the other two MBSs on the domains are defined as

$$\gamma_3 = -ie^{i\frac{\theta_L}{2}}\hat{c}_3 + ie^{-i\frac{\theta_L}{2}}\hat{c}_3^\dagger, \quad \gamma_4 = e^{i\frac{\theta_R}{4}}\hat{c}_2 + e^{-i\frac{\theta_R}{2}}\hat{c}_4^\dagger \tag{S2}$$

where $\hat{c}_{3,4}$ are the local fermion operators at the domain walls. For each of the four MBSs, there is a partner, with which the local fermion operator is reconstructed as

$$\begin{aligned}\hat{c}_1 &= e^{-i\frac{\theta_L}{2}}(\gamma_1 + i\gamma_1')/2, \quad \hat{c}_2 = e^{-i\frac{\theta_R}{2}}(\gamma_2' + i\gamma_2)/2\\ \hat{c}_3 &= e^{-i\frac{\theta_L}{2}}(\gamma_3' + i\gamma_3)/2, \quad \hat{c}_4 = e^{-i\frac{\theta_R}{2}}(\gamma_4 + i\gamma_4')/2.\end{aligned} \tag{S3}$$

It is noted that $\gamma'_{1,2,3,4}$ correspond to excitations above the superconducting energy gap, and thus are irrelevant to low-energy physics.

The two MBSs in each segment have a small coupling due to the finite wire length, resulting in the coupling Hamiltonian given first by Kitaev[1]

$$\mathcal{H}_\delta = i\delta_L \gamma_1 \gamma_3 + i\delta_R \gamma_4 \gamma_2. \tag{S4}$$

The coupling energy $\delta_{L,R}$ is considered as a small parameter in the present work. The two segments are coupled at the junction through the electron tunneling

$$\begin{aligned}\mathcal{H}_J &= J(\hat{c}_1^\dagger \hat{c}_2 + \hat{c}_2^\dagger \hat{c}_1) \\ &= Je^{\frac{i\phi}{2}}(\gamma_1\gamma_2' - i\gamma_1'\gamma_2' + i\gamma_1\gamma_2 + \gamma_1'\gamma_2)/4 \\ &+ Je^{-\frac{i\phi}{2}}(\gamma_2'\gamma_1 - i\gamma_2\gamma_1 + i\gamma_2'\gamma_1' + \gamma_2\gamma_1')/4 \\ &\approx iJ\cos\frac{\phi}{2}\gamma_1\gamma_2,\end{aligned} \tag{S5}$$

with $\phi = \theta_L - \theta_R$ the phase difference across the junction, where all excitations above the superconducting gap are neglected presuming that the system is kept at sufficiently low temperature. This fractional Josephson energy was derived by Alicea *et al*[2]. These two energies are contributed only from MBSs, and are summarized in Eq. (1) in the main text

$$\mathcal{H}_M = \mathcal{H}_\delta + \mathcal{H}_J. \tag{S6}$$

The quantum dot (QD) is prepared in a way that there is only one energy level:

$$\mathcal{H}_D = \epsilon d^\dagger d, \tag{S7}$$

where $d^\dagger$ is the electron creation operator and ϵ is the occupation energy.

When the QD is connected to the junction in a tunneling way one has an additional contribution to total Hamiltonian,

$$\begin{aligned}\mathcal{H}_T &= (T_1^*\hat{c}_1^\dagger d + T_1 d^\dagger \hat{c}_1) + (T_2\hat{c}_2^\dagger d + T_2^* d^\dagger \hat{c}_2) \\ &= \left[T_1^* e^{\frac{i\theta_L}{2}}(\gamma_1 - i\gamma_1')d + T_1 d^\dagger e^{-\frac{i\theta_L}{2}}(\gamma_1 + i\gamma_1')\right] \\ &+ \left[T_2 e^{\frac{i\theta_R}{2}}(\gamma_2' - i\gamma_2)d + T_2^* d^\dagger e^{-\frac{i\theta_R}{2}}(\gamma_2' + i\gamma_2)\right] \\ &\approx \left[T_1^* e^{\frac{i\theta_L}{2}}\gamma_1 d + T_1 e^{-\frac{i\theta_L}{2}} d^\dagger \gamma_1\right] + \left[-iT_2 e^{\frac{i\theta_R}{2}}\gamma_2 d + iT_2^* e^{-\frac{i\theta_R}{2}} d^\dagger \gamma_2\right] \\ &= (T_1 d^\dagger - T_1^* d)\gamma_1 + i(T_2 d^\dagger + T_2^* d)\gamma_2,\end{aligned} \tag{S8}$$

where high-energy excitations are neglected as above, and the superconducting phases θ_L and θ_R are included into the tunneling matrix elements T_1 and T_2.

Now we formulate the total Hamiltonian into the most convenient matrix form. For this purpose, we redefine fermionic operators,

$$f_1^\dagger = (\gamma_1 - i\gamma_2)/2, \quad f_2^\dagger = (\gamma_4 - i\gamma_3)/2. \tag{S9}$$

The MBS operators can be rewritten as

$$\begin{aligned} \gamma_1 &= f_1^\dagger + f_1, \quad \gamma_2 = i(f_1^\dagger - f_1), \\ \gamma_3 &= i(f_2^\dagger - f_2), \quad \gamma_4 = f_2^\dagger + f_2, \end{aligned} \tag{S10}$$

and the Hamiltonian is given by

$$\mathcal{H}_M = J\cos\frac{\phi}{2}(2f_1^\dagger f_1 - 1) + (\delta_L + \delta_R)(f_2^\dagger f_1 + f_1^\dagger f_2) + (\delta_L - \delta_R)(f_2^\dagger f_1^\dagger + f_1 f_2), \tag{S11}$$

and

$$\begin{aligned} \mathcal{H}_T &= (T_1 d^\dagger - T_1^* d)\gamma_1 + i(T_2 d^\dagger + T_2^* d)\gamma_2, \qquad \text{(S12)} \\ &= (T_1 d^\dagger - T_1^* d)(f_1^\dagger + f_1) - (T_2 d^\dagger + T_2^* d)(f_1^\dagger - f_1) \\ &= (T_2 - T_1) f_1^\dagger d^\dagger + (T_2^* - T_1^*) d f_1 + (T_2 + T_1) d^\dagger f_1 + (T_2^* + T_1^*) f_1^\dagger d \\ &= T_a f_1^\dagger d^\dagger + T_a^* d f_1 + T_b d^\dagger f_1 + T_b^* f_1^\dagger d \end{aligned}$$

with $T_a = T_2 - T_1$ and $T_b = T_2 + T_1$

Using the fermionic states, a low-energy state of the system can be described by

$$\begin{aligned} |G\rangle &= P_1|0\rangle + P_2 f_1^\dagger d^\dagger|0\rangle + P_3 f_2^\dagger f_1^\dagger|0\rangle + P_4 f_2^\dagger d^\dagger|0\rangle \\ &+ P_5 f_1^\dagger|0\rangle + P_6 d^\dagger|0\rangle + P_7 f_2^\dagger|0\rangle + P_8 f_2^\dagger f_1^\dagger d^\dagger|0\rangle, \end{aligned} \tag{S13}$$

and the Hamiltonian is recasted into a 8x8 matrix,

$$\begin{pmatrix} -J\cos\frac{\phi}{2} & T_a^* & \delta_L-\delta_R & 0 & 0 & 0 & 0 & 0 \\ T_a & J\cos\frac{\phi}{2}+\epsilon & 0 & \delta_L+\delta_R & 0 & 0 & 0 & 0 \\ \delta_L-\delta_R & 0 & J\cos\frac{\phi}{2} & T_b^* & 0 & 0 & 0 & 0 \\ 0 & \delta_L-\delta_R & T_b & -J\cos\frac{\phi}{2}+\epsilon & 0 & 0 & 0 & 0 \\ 0 & 0 & 0 & 0 & J\cos\frac{\phi}{2} & T_b^* & \delta_L+\delta_R & 0 \\ 0 & 0 & 0 & 0 & T_b & -J\cos\frac{\phi}{2}+\epsilon & 0 & \delta_L-\delta_R \\ 0 & 0 & 0 & 0 & \delta_L+\delta_R & 0 & -J\cos\frac{\phi}{2} & T_a^* \\ 0 & 0 & 0 & 0 & 0 & \delta_L-\delta_R & T_a & J\cos\frac{\phi}{2}+\epsilon \end{pmatrix}. \tag{S14}$$

It is block-diagonalized into two 4x4 matrices, for even- and odd-parity subspace respectively. When QD is coupled to only one of the junction MBSs, namely $T_1 = 0$, one has $T_a = T_b = T$ in the above matrix, which results in Eq. (3) in the main text. In a general case, the matrix elements (1,2) and (3,4) are not equal to each other. However, it is clear that as far as the two tunneling couplings are controlled by the same frequency, the shape of spectra of Rabi oscillations presented in Fig. 3 in the main text remains the same, which exhibits the fractional Josephson relation, with heights changed slightly according to second-order perturbations. In the special case where the two couplings become exactly the same, matrix elements (1,2) or (3,4) become zero, and one of the two curves in Fig. 3 in the main text fades away proportional to the coupling difference. In this case, the odd-parity subspace contributes complementally the other curve as can be seen in the matrix in Eq. (S14).

Floquet theory

The Hamiltonian $\mathcal{H}(t)$ in Eq. (3) in main text is periodic with a period of $T = 2\pi/\Omega$. Its Fourier transformation is given by

$$\mathcal{H}(t) = \sum_m \mathcal{H}_m e^{im\Omega t}, \tag{S15}$$

with

$$\mathcal{H}_0 = \begin{pmatrix} -J\cos\frac{\phi}{2} & T_0 & \delta_\mathrm{L}-\delta_\mathrm{R} & 0 \\ T_0 & J\cos\frac{\phi}{2}+\epsilon & 0 & \delta_\mathrm{L}+\delta_\mathrm{R} \\ \delta_\mathrm{L}-\delta_\mathrm{R} & 0 & J\cos\frac{\phi}{2} & T_0 \\ 0 & \delta_\mathrm{L}+\delta_\mathrm{R} & T_0 & -J\cos\frac{\phi}{2}+\epsilon \end{pmatrix}, \tag{S16}$$

and

$$\mathcal{H}_1 = \mathcal{H}_{-1} = \begin{pmatrix} 0 & T_1 & 0 & 0 \\ T_1 & 0 & 0 & 0 \\ 0 & 0 & 0 & T_1 \\ 0 & 0 & T_1 & 0 \end{pmatrix}, \tag{S17}$$

with all other components zero.

Floquet theorem, a time version of Bloch theorem in crystal with periodic potential in space[3], states that the wave functions governed by this time-periodic Hamiltonian is given by

$$\Psi(t) \equiv [P_1(t), P_2(t), P_3(t), P_4(t)]^{\mathrm{t}} = \psi(t)e^{-iQt}, \tag{S18}$$

with $\psi(t) = \psi(t + 2\pi/\Omega)$ where Q is to be determined. Now we perform Fourier transform on the wave function with period $T = 2\pi/\Omega$,

$$\psi(t) = \sum_n \psi_n e^{in\Omega t}, \tag{S19}$$

where ψ_n is a vector with 4 components $\psi_{n,\alpha}$.

We look back to Eq. (3) in main text which can be written in a compact form

$$i\frac{d}{dt}\Psi(t) = \mathcal{H}(t)\Psi(t), \tag{S20}$$

where $\hbar = 1$ is understood. Plugging the Fourier transformations into the above equation, it is reformulated into

$$\sum_n (Q - n\Omega)\psi_{n,\alpha}e^{in\Omega t} = \sum_{m,l,\beta} \mathcal{H}_{m,\alpha,\beta}\psi_{l,\beta}e^{i(m+l)\Omega t}, \tag{S21}$$

where α, β indicate the elements of each 4x4 matrix $\mathcal{H}_m$. It is clear that one should have

$$Q\psi_{n,\alpha} = \sum_{l,\beta} \left(\mathcal{H}_{n-l,\alpha,\beta} + n\Omega I_{\alpha,\beta}\right)\psi_{l,\beta}, \tag{S22}$$

where $I_{\alpha,\beta}$ is the element of the 4x4 unit matrix. We now have a static eigen-problem, with eigen value Ω and eigen function $\psi_{n,\alpha}$. Defining the eigen vector $\tilde{\psi} = (..., \psi_{-1}^{\mathrm{t}}, \psi_0^{\mathrm{t}}, \psi_1^{\mathrm{t}}, ...)^{\mathrm{t}}$,

the resultant Floquet Hamiltonian $\mathcal{H}_{\mathrm{f}}$ should be

$$\begin{pmatrix} \cdot & \cdot & \cdot & \cdot & \cdot & \cdot & \cdot \\ \cdot & \mathcal{H}_0 - 2\Omega\hat{I} & \mathcal{H}_1 & 0 & 0 & 0 & \cdot \\ \cdot & \mathcal{H}_1 & \mathcal{H}_0 - \Omega\hat{I} & \mathcal{H}_1 & 0 & 0 & \cdot \\ \cdot & 0 & \mathcal{H}_1 & \mathcal{H}_0 & \mathcal{H}_1 & 0 & \cdot \\ \cdot & 0 & 0 & \mathcal{H}_1 & \mathcal{H}_0 + \Omega\hat{I} & \mathcal{H}_1 & \cdot \\ \cdot & 0 & 0 & 0 & \mathcal{H}_1 & \mathcal{H}_0 + 2\Omega\hat{I} & \cdot \\ \cdot & \cdot & \cdot & \cdot & \cdot & \cdot & \cdot \end{pmatrix}. \tag{S23}$$

The problem of solving time-dependent Schröding equation (S19) now is reduced to a static eigen problem. The price one has to pay is to expand a 4x4 matrix into an matrix with infinite dimensions. Now we show how the Floquet Hamiltonian determines the quantum evolution of the system. We define a Floquet state $|n, \alpha\rangle$ by $\psi_{n,\alpha}$ with α denoting one of the quantum states $|S_1\rangle, |S_2\rangle, |S_3\rangle$ and $|S_4\rangle$ and n the Fourier component. We notice that the eigen-value of the Floquet Hamiltonian has a periodic property, that is, if Q is a eigen value, then $Q + n\Omega$ is also an eigen value. This periodic property comes from the block-diagonal nature of the Floquet hamiltonian. Therefore, we can label eigen values by,

$$Q_{n,\gamma} = q_\gamma + n\Omega, \tag{S24}$$

where

$$q_\gamma = Q_{0,\gamma} \tag{S25}$$

denote the four eigen values with smallest absolute values. Meanwhile, the eigen functions are also periodic, that is, for any eigen vector $|Q_{n,\gamma}\rangle$ corresponding to eigenvalue $Q_{n,\gamma}$, we have

$$\langle l, \alpha | Q_{n,\gamma}\rangle = \langle l + m, \alpha | Q_{n+m,\gamma}\rangle. \tag{S26}$$

It is clear that despite the infinite dimensionless of the Floquet Hamiltonian $\mathcal{H}_{\mathrm{f}}$, there are actually only four independent eigen-values and eigen-vectors. We can extract all information and reconstruct the time-dependent wave-function with the four eigen values q_γ and the four corresponding eigen-vectors $|q_\gamma\rangle$.

Now we consider the transition probability between two states $|\alpha\rangle$ and $|\beta\rangle$ as a function of time. The evolution operator between these two states is given by[4]

$$\hat{U}_{\beta,\alpha}(t;t_0) = \sum_{n,\gamma} P_\gamma \psi_{n,\beta,\gamma}(t), \tag{S27}$$

with $P_\gamma = \sum_n \psi^*_{n,\alpha,\gamma} e^{-in\Omega t_0} e^{iQ_\gamma t_0}$ provided that the system starts from the state $|\alpha\rangle$ at time t_0. Taking advantage of Eq. (S25), one can prove

$$\hat{U}_{\beta,\alpha}(t;t_0) = \sum_n \langle n,\beta| e^{-i\mathcal{H}_{\mathrm{f}}(t-t_0)} |0,\alpha\rangle e^{in\Omega t}. \tag{S28}$$

Considering that the transition probability should be an average of t_0 with $t - t_0$ constant, since the initial phase of the system is not well defined, we obtain the transition probability

$$P_{\alpha->\beta} = \sum_n \left| \langle n,\beta| e^{-i\hat{H}_f(t-t_0)} |0,\alpha\rangle \right|^2. \tag{S29}$$

Now the transition probability between the two states governed by the time-periodic Hamiltonian is given in terms of the summation on transition probabilities between Floquet states governed by time-independent Floquet Hamiltonian. For short-range couplings between blocks associated with different photons in Floquet Hamiltonian, namely for ac driving with a few Fourier components, the number of terms in the above summation is limited.

Rabi oscillations

Next we analyze possible Rabi oscillatons in the system. In a resonant oscillation, two Floquet states become degenerate in energy, which can be captured by a 2x2 matrix for these two states, while all other matrix elements are taken into account as perturbations. These perturbations slightly shift the energies of the two Floquet states, and modulate the matrix element between them as well. The effective 2x2 matrix will provide the oscillation conditions.

Let us first discuss the oscillation between $|S_1\rangle$ and $|S_2\rangle$. There is a contribution from the Floquet states $|0, S_1\rangle$ and $|-1, S_2\rangle$ with the bare 2x2 matrix

$$\mathcal{H} = \begin{pmatrix} -J\cos\frac{\phi}{2} & T_1 \\ T_1 & J\cos\frac{\phi}{2} + \epsilon - \Omega \end{pmatrix}. \tag{S30}$$

It is obvious that there is a resonant Rabi oscillation around $\Omega \approx 2J\cos\frac{\phi}{2} + \epsilon$. We now consider contributions from other states in terms of the second-order perturbations. Since

$|0,S1\rangle$ has matrix elements with $|0,S_2\rangle$, $|0,S_3\rangle$, $|1,S_2\rangle$, its energy is shifted by

$$
\begin{aligned}
E_{-} &= -\frac{T_0^2}{2J\cos\frac{\phi}{2}+\epsilon} - \frac{(\delta_L-\delta_R)^2}{2J\cos\frac{\phi}{2}} - \frac{T_1^2}{4J\cos\frac{\phi}{2}+2\epsilon} \\
&= -\frac{2T_0^2+T_1^2}{4J\cos\frac{\phi}{2}+2\epsilon} - \frac{(\delta_L-\delta_R)^2}{2J\cos\frac{\phi}{2}}.
\end{aligned}
\tag{S31}
$$

Similarly, since $|-1,S2\rangle$ has matrix elements with $|-1,S_1\rangle$, $|-1,S_4\rangle$, $|-2,S_1\rangle$, its energy shift is given by

$$
E_{+} = \frac{2T_0^2+T_1^2}{4J\cos\frac{\phi}{2}+2\epsilon} + \frac{(\delta_L+\delta_R)^2}{2J\cos\frac{\phi}{2}}.
\tag{S32}
$$

Noticing that there is no two-step virtual hopping process between these two states, the off-diagonal elements are not modified within the second-order perturbation. In this way we arrive at the effective Hamiltonian Eq. (10) in main text. Considering the transformation between $|0,S_1\rangle$ and $|1,S_2\rangle$ in the same way, the condition for the Rabi oscillation between $|S_1\rangle$ and $|S_2\rangle$ is given by Eq. (12) in main text. Rabi oscillation between $|S_3\rangle$ and $|S_4\rangle$ can be derived similarly.

Rabi oscillation takes place between $|S_1\rangle$ and $|S_4\rangle$, for which we should look at the Floquet states $|0,S_1\rangle$ and $|-1,S_4\rangle$. The bare 2x2 matrix is

$$
\mathcal{H} = \begin{pmatrix} -J\cos\frac{\phi}{2} & 0 \\ 0 & -J\cos\frac{\phi}{2}+\epsilon-\Omega \end{pmatrix}.
\tag{S33}
$$

These two states are connected with each other through $|-1,S_2\rangle$ and $|0,S_3\rangle$, which produce nonzero off-diagonal elements through the second-order perturbation,

$$
\mathcal{H}_{14} = \sum_{n,\alpha} \frac{\langle 0,S1|\hat{H}|n,\alpha\rangle\langle n,\alpha|\hat{H}|-1,S4\rangle}{\langle 0,S1|\hat{H}|0,S1\rangle - \langle n,\alpha|\hat{H}|n,\alpha\rangle}
\tag{S34}
$$

$$
= \frac{T_1(\delta_L-\delta_R)}{-J\cos\frac{\phi}{2}-J\cos\frac{\phi}{2}} + \frac{(\delta_L+\delta_R)T_1}{-J\cos\frac{\phi}{2}-J\cos\frac{\phi}{2}}
\tag{S35}
$$

$$
= -\frac{T_1\delta_L}{J\cos\frac{\phi}{2}}.
\tag{S36}
$$

The energy shift for state $|0,S_1\rangle$ is

$$
\begin{aligned}
E'_{-} &= -\frac{T_0^2}{2J\cos\frac{\phi}{2}+\epsilon} - \frac{(\delta_L-\delta_R)^2}{2J\cos\frac{\phi}{2}} \\
&\quad -\frac{T_1^2}{2J\cos\frac{\phi}{2}+2\epsilon} - \frac{T_1^2}{2J\cos\frac{\phi}{2}},
\end{aligned}
\tag{S37}
$$

and for $|-1, S_4\rangle$

$$E'_{+} = \frac{T_0^2}{2J\cos\frac{\phi}{2}+\epsilon} + \frac{(\delta_L+\delta_R)^2}{2J\cos\frac{\phi}{2}} + \frac{T_1^2}{2J\cos\frac{\phi}{2}+2\epsilon} + \frac{T_1^2}{2J\cos\frac{\phi}{2}}. \tag{S38}$$

We then arrive at the effective 2x2 matrix within the second-order perturbation

$$\mathcal{H} = \begin{pmatrix} -J\cos\frac{\phi}{2}+E'_{-} & \frac{T_1\delta_L}{J\cos\phi/2} \\ \frac{T_1\delta_L}{J\cos\phi/2} & -J\cos\frac{\phi}{2}+\epsilon-\Omega+E'_{+} \end{pmatrix}. \tag{S39}$$

A Rabi oscillation appears at $\Omega = \epsilon + E'_{+} - E'_{-}$, with frequency of $T_1\delta_L/(J\cos\frac{\phi}{2})$.

We notice two interesting properties of this Rabi oscillation. First, its frequency is smaller than the others discussed above by order of δ_L/J, since this oscillation is driven by second-order hopping processes. Second, this frequency contains only δ_L, while δ_R is missing. This is related to the structure of setup where QD is connected to junction MBS on the right segment (see Fig. 1 of main text). The transformation from $|S_1\rangle$ to $|S_4\rangle$ is contributed from four possible processes. For the first two processes, a) an electron jumps from the junction to QD and then another electron jumps from the left segment to junction

$$(\delta_L\gamma_1\gamma_3)(iTd^\dagger\gamma_2), \tag{S40}$$

and b) an electron jumps from the left segment to junction and then jumps to QD

$$(iTd^\dagger\gamma_2)(\delta_L\gamma_1\gamma_3). \tag{S41}$$

The total contribution is $2iT\delta_L d^\dagger\gamma_2\gamma_1\gamma_3$. Similarly, the other two processes include an electron coming from right segment, which are described by

$$(\delta_R\gamma_4\gamma_2)(iTd^\dagger\gamma_2), \tag{S42}$$

and

$$(iTd^\dagger\gamma_2)(\delta_R\gamma_4\gamma_2). \tag{S43}$$

However, in contrast to the previous case, the two processes interfere with each other destructively

$$iT\delta_R(d^\dagger\gamma_2\gamma_4\gamma_2 + \gamma_4\gamma_2 d^\dagger\gamma_2) = 0, \tag{S44}$$

and make a vanishing contribution to the transformation. In other words, the oscillation between $|S_1\rangle$ to $|S_4\rangle$ only involves electron hopping from the left segment, thus δ_R is missing in the Rabi frequency. One can understand the Rabi oscillation between $|S_2\rangle$ to $|S_3\rangle$ in the same way.

Finally, we should note that the above discussions in terms of 2x2 matrix are based on perturbation approach, and therefore are not valid when the energy shift and off-diagonal elements are large. Especially, when $\cos\phi/2 = 0$ or $2J\cos\phi/2 \pm \epsilon = 0$, denominators in energy shifts and coupling elements become zero. These cases correspond to an additional energy degeneracy besides the two states under concern, where two sets of Rabi oscillatons take place simultaneously as can be seen in Fig. 3 of main text.

Multi-state oscillation

Here we analyze the crossing point of in Fig. 3 at $\Phi = \Phi_0/2$ and $\Omega = \epsilon$ within the Floquet formalism. At this special point, the four Floquet states $|0, S_1\rangle$, $|-1, S_2\rangle$, $|0, S_3\rangle$, and $|-1, S_4\rangle$ with approximately zero energy are involved into resonant oscillation. The unperturbed Hamiltonian is given by

$$\mathcal{H} = \begin{pmatrix} 0 & T_1 & \delta_\mathrm{L} - \delta_\mathrm{R} & 0 \\ T_1 & 0 & 0 & \delta_\mathrm{L} + \delta_\mathrm{R} \\ \delta_\mathrm{L} - \delta_\mathrm{R} & 0 & 0 & T_1 \\ 0 & \delta_\mathrm{L} + \delta_\mathrm{R} & T_1 & 0 \end{pmatrix}, \tag{S45}$$

Interchanging the order of the basis state $|-1, S_2\rangle$ and $|0, S_3\rangle$, the matrix is transformed to,

$$\mathcal{H} = \begin{pmatrix} 0 & \delta_\mathrm{L} - \delta_\mathrm{R} & T_1 & 0 \\ \delta_\mathrm{L} - \delta_\mathrm{R} & 0 & 0 & T_1 \\ T_1 & 0 & 0 & \delta_\mathrm{L} + \delta_\mathrm{R} \\ 0 & T_1 & \delta_\mathrm{L} + \delta_\mathrm{R} & 0 \end{pmatrix}. \tag{S46}$$

It is clear that oscillations should appear on the QD, since the diagonal terms are all equivalent to zero. Now we show that the oppilation is actually a combination of Rabi oscillations with two slightly different frequencies. We construct "bonding" states

$$\begin{aligned} |S_{13}^{+}\rangle &\equiv \frac{1}{\sqrt{2}}(|0, S_1\rangle + |0, S_3\rangle), \\ |S_{24}^{+}\rangle &\equiv \frac{1}{\sqrt{2}}(|-1, S_2\rangle + |-1, S_4\rangle), \end{aligned} \tag{S47}$$

and "anti-bonding" states

$$|S_{13}^{-}\rangle \equiv \frac{1}{\sqrt{2}}(|0,S_1\rangle - |0,S_3\rangle),$$
$$|S_{24}^{-}\rangle \equiv \frac{1}{\sqrt{2}}(|-1,S_2\rangle - |-1,S_4\rangle). \tag{S48}$$

The Hamiltonian is transformed into

$$\mathcal{H} = \begin{pmatrix} \delta_{\mathrm{L}}-\delta_{\mathrm{R}} & 0 & T_1 & 0 \\ 0 & -\delta_{\mathrm{L}}+\delta_{\mathrm{R}} & 0 & T_1 \\ T_1 & 0 & \delta_{\mathrm{L}}+\delta_{\mathrm{R}} & 0 \\ 0 & T_1 & 0 & -\delta_{\mathrm{L}}-\delta_{\mathrm{R}} \end{pmatrix} \tag{S49}$$

in the new basis $|S_{13}^{+}\rangle$, $|S_{13}^{-}\rangle$, $|S_{24}^{+}\rangle$, $|S_{24}^{-}\rangle$. For example, the matrix elements in the first row are calculated as,

$$\langle S_{13}^{+}|\mathcal{H}|S_{13}^{+}\rangle = \frac{1}{2}[\langle S_1|\mathcal{H}|S_1\rangle + \langle S_1|\mathcal{H}|S_3\rangle + \langle S_3|\mathcal{H}|S_1\rangle + \langle S_3|\mathcal{H}|S_3\rangle]$$
$$= \frac{1}{2}\left[0 + (\delta_L - \delta_R) + (\delta_L - \delta_R) + 0\right] = \delta_L - \delta_R, \tag{S50}$$

$$\langle S_{13}^{+}|\mathcal{H}|S_{13}^{-}\rangle = \frac{1}{2}\left[\langle S_1|\mathcal{H}|S_1\rangle - \langle S_1|\mathcal{H}|S_3\rangle + \langle S_3|\mathcal{H}|S_1\rangle - \langle S_3|\mathcal{H}|S_3\rangle\right]$$
$$= \frac{1}{2}\left[0 - (\delta_L - \delta_R) + (\delta_L - \delta_R) + 0\right] = 0, \tag{S51}$$

$$\langle S_{13}^{+}|\mathcal{H}|S_{24}^{+}\rangle = \frac{1}{2}\left[\langle S_1|\mathcal{H}|S_2\rangle + \langle S_1|\mathcal{H}|S_4\rangle + \langle S_3|\mathcal{H}|S_2\rangle + \langle S_3|\mathcal{H}|S_4\rangle\right]$$
$$= \frac{1}{2}\left[T_1 + 0 + 0 + T_1\right] = T_1, \tag{S52}$$

$$\langle S_{13}^{+}|\mathcal{H}|S_{24}^{-}\rangle = \frac{1}{2}\left[\langle S_1|\mathcal{H}|S_2\rangle - \langle S_1|\mathcal{H}|S_4\rangle + \langle S_3|\mathcal{H}|S_2\rangle - \langle S_3|\mathcal{H}|S_4\rangle\right]$$
$$= \frac{1}{2}\left[T_1 + 0 + 0 - T_1\right] = 0, \tag{S53}$$

and all other elements can be obtained similarly. Now the Hamiltonian can be block diagonalized by reordering the basis as $|S_{13}^{+}\rangle$, $|S_{24}^{+}\rangle$, $|S_{13}^{-}\rangle$, $|S_{24}^{-}\rangle$,

$$\mathcal{H} = \begin{pmatrix} \delta_{\mathrm{L}}-\delta_{\mathrm{R}} & T_1 & 0 & 0 \\ T_1 & \delta_{\mathrm{L}}+\delta_{\mathrm{R}} & 0 & 0 \\ 0 & 0 & -\delta_{\mathrm{L}}+\delta_{\mathrm{R}} & T_1 \\ 0 & 0 & T_1 & -\delta_{\mathrm{L}}-\delta_{\mathrm{R}} \end{pmatrix}. \tag{S54}$$

The physics can be understood in a more transparent way. For $\Phi = \Phi_0/2$, there is no coupling among the two MBSs γ_1 and γ_2. It is then better to describe the system by parity state of electron number on the left segment formed by γ_1 and γ_3, and that on the right segment formed by γ_4 and γ_2. It is not difficult to see that the bonding state $|S_{13}^{+}\rangle$ corresponds to even parity states on both left and right segments, while $|S_{24}^{+}\rangle$ corresponds to even parity on the left segment while odd parity on the right segment, and the two states are coupled via T_1 which changes the parity of the right segment using QD. The same applies for the two anti-bonding states. The absence of direct coupling between the two blocks is due to the structure of system setup where QD is only connected to the right segment.

Now we consider the energy shifts for these 'bonding' and 'anti-bonding' states with the second-order perturbation treatment. Since $\epsilon \gg T_0 \gg T_1, \delta$, dominant contributions should come from virtual processes from Floquet states in the same block. For $|S_{13}^{+}\rangle$, the two Floquet states $|0, S_2\rangle$ and $|0, S_4\rangle$ with the same energy ϵ and same coupling matrix element

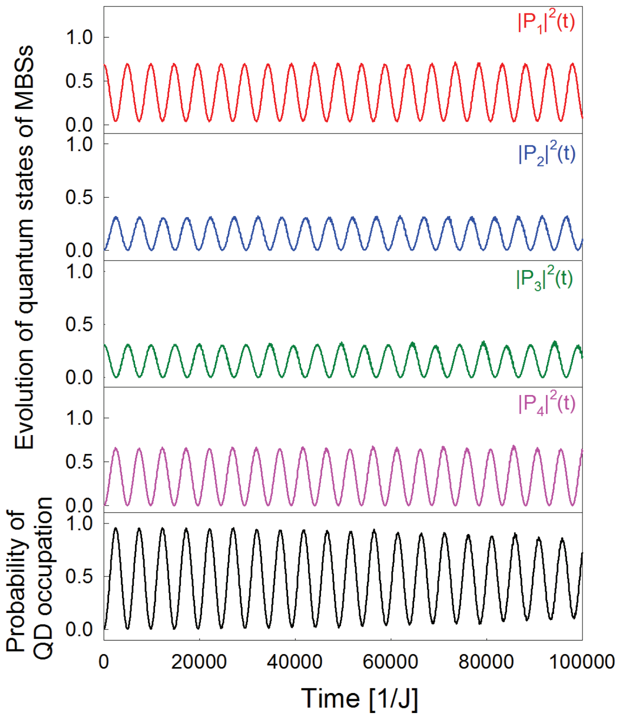

FIG. S1. (Color online). Time evolution of the quantum state of MBSs and the QD occupation probability with initial condition of $|P_1|^2 = 0.7$ and $|P_3|^2 = 0.3$ for $\Phi = 0.1\Phi_0$ and $\Omega = \epsilon = 0.399J$, and other parameters are the same as in Fig. 2 in the main text.

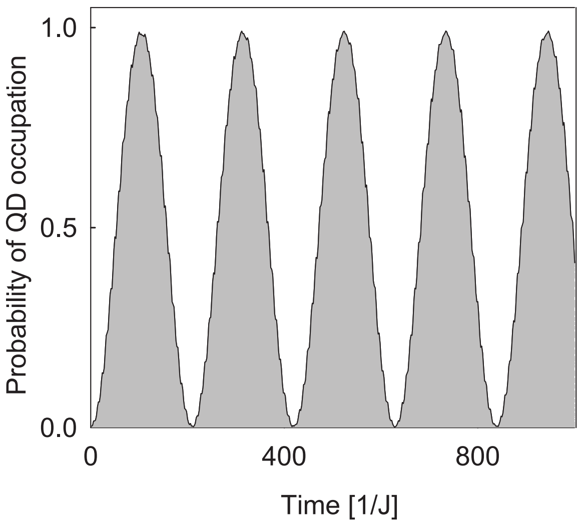

FIG. S2. (Color online). Envelop function (black curve) for repeated experimental measurements on the occupation probability of QD starting from an empty initial state. The parameters are the same as the Fig. 2a.

T_0 with $|S_{13}^{+}\rangle$ contribute an energy shift

$$E_1^{''} = \frac{2T_0^2}{\delta_{\rm L} - \delta_{\rm R} - \epsilon}. \tag{S55}$$

For $|S_{24}^{+}\rangle$, the two Floquet states $|-1, S_1\rangle$ and $|-1, S_3\rangle$ with energy $-\epsilon$ and coupling matrix element T_0 with $|S_{24}^{+}\rangle$ contribute an energy shift

$$E_2^{''} = \frac{2T_0^2}{\delta_{\rm L} + \delta_{\rm R} + \epsilon}. \tag{S56}$$

Similarly, the anti-bonding state $|S_{13}^{-}\rangle$ is connected to $|0, S_2\rangle$ and $|0, S_4\rangle$ with coupling matrix elements T_0 and $-T_0$ respectively, getting an energy shift

$$E_3^{"} = \frac{2T_0^2}{-\delta_{\rm L} + \delta_{\rm R} - \epsilon}, \tag{S57}$$

and $|S_{24}^{-}\rangle$ is connected to $|-1, S_1\rangle$ and $|-1, S_3\rangle$ with coupling matrix elements T_0 and $-T_0$ respectively, getting an energy shift

$$E_4^{"} = \frac{2T_0^2}{-\delta_{\rm L} - \delta_{\rm R} + \epsilon}. \tag{S58}$$

The matrix up to the second-order perturbation is given as,

$$\mathcal{H} = \begin{pmatrix} \delta_{\rm L} - \delta_{\rm R} + E_1^{"} & T_1 & 0 & 0 \\ T_1 & \delta_{\rm L} + \delta_{\rm R} + E_2^{"} & 0 & 0 \\ 0 & 0 & -\delta_{\rm L} + \delta_{\rm R} + E_3^{"} & T_1 \\ 0 & 0 & T_1 & -\delta_{\rm L} - \delta_{\rm R} + E_4^{"} \end{pmatrix}. \tag{S59}$$

There is a Rabi oscillation between the two bonding states with frequency

$$\omega_1 = \sqrt{(2\delta_R + E_2^{"} - E_1^{"})^2 + T_1^2}, \tag{S60}$$

and between the two anti-bonding states with frequency

$$\omega_2 = \sqrt{(2\delta_R + E_3^{"} - E_4^{"})^2 + T_1^2}. \tag{S61}$$

It is noticed that the oscillations are not full (see Eq. (11) in main text), and thus the two frequencies are not equal even though the off-diagonal coupling elements in the two blocks are both T_1.

Results based on numerical simulation

Besides the analytic treatment based on Floquet theorem, we also perform numerical calculation by integrating directly the time-dependent Schrödinger equation (3) in main text. The results are summarized in Fig. 3 of main text, which are in good agreement with analytic results, except for the region around the crossing points of curves where the simple treatment of 2x2 matrix breaks down.

In numerical simulations, we start from a quantum state with empty QD with $|P_1|^2 = |P_3|^2 = 1/2$. The strength of Rabi oscillation given by color scale in Fig. 3 in main text is measured by the difference in occupation probability of electron on QD during the Rabi oscillation.

We now provide some detailed numerical results on the dynamics of system when the driving frequency is tuned to $\Omega = \epsilon = 0.399J$, corresponding to the horizontal line in Fig. 3. As shown explicitly in Fig. S1 for $\Phi = 0.1\Phi_0$, oscillation between $|S_1\rangle$ and $|S_4\rangle$ and that between $|S_2\rangle$ and $|S_3\rangle$ take place simultaneously. We particularly note that the period of these oscillations is much longer than those in Figs. 2, in agreement with Eq. (S39).

Last we discuss some details relevant to experiments. In the present system, we initialize the system by measuring the electron occupation on QD. In theoretical analyses we obtain

the Rabi oscillation patterns for an initial condition $P_1(0) = P_3(0) = 1/\sqrt{2}$. However, one cannot control the coefficients $P_1(0)$ and $P_3(0)$ in real experiments, and they vary between 0 and 1 in repeated measurements with $P_1^2(0) + P_3^2(0) = 1$. The measured QD occupation probability should then display a group of curves associated with various pairs of $P_1(0)$ and $P_3(0)$, which fall into the shadowed region in Fig. S2.

[1] A. Kitaev, *Phys. Usp.* **44**, 131 (2001).

[2] J. Alicea, Y. Oreg, G. Refael, F. von Oppen and M. P. A. Fisher, *Nature Phys.* **7**, 412 (2011).

[3] G. D. Mahan, *Condensed matter in a nutshell* (Princton University Press, 2011).

[4] J. H. Shirley, *Phys. Rev.* **B138**, 979 (1965).
